# Optomyography-based sensing of facial expression derived arousal and valence in adults with depression

**DOI:** 10.3389/fpsyt.2023.1232433

**Published:** 2023-08-08

**Authors:** M. John Broulidakis, Ivana Kiprijanovska, Liberty Severs, Simon Stankoski, Martin Gjoreski, Ifigeneia Mavridou, Hristijan Gjoreski, Sophia Cox, Daisy Bradwell, James M. Stone, Charles Nduka

**Affiliations:** ^1^Emteq Ltd., Brighton, United Kingdom; ^2^Faculty of Informatics, Università della Svizzera italiana, Lugano, Switzerland; ^3^Ss. Cyril and Methodius University in Skopje (UKIM), Skopje, North Macedonia; ^4^Brighton and Sussex Medical School, University of Sussex, Brighton, United Kingdom; ^5^Queen Victoria Hospital, East Grinstead, United Kingdom

**Keywords:** optomyography, depression, wearable computing, digital phenotyping, facial expressions

## Abstract

**Background:**

Continuous assessment of affective behaviors could improve the diagnosis, assessment and monitoring of chronic mental health and neurological conditions such as depression. However, there are no technologies well suited to this, limiting potential clinical applications.

**Aim:**

To test if we could replicate previous evidence of hypo reactivity to emotional salient material using an entirely new sensing technique called optomyography which is well suited to remote monitoring.

**Methods:**

Thirty-eight depressed and 37 controls (≥18, ≤40 years) who met a research diagnosis of depression and an age-matched non-depressed control group. Changes in facial muscle activity over the brow (corrugator supercilli) and cheek (zygomaticus major) were measured whilst volunteers watched videos varying in emotional salience.

**Results:**

Across all participants, videos rated as subjectively positive were associated with activation of muscles in the cheek relative to videos rated as neutral or negative. Videos rated as subjectively negative were associated with brow activation relative to videos judged as neutral or positive. Self-reported arousal was associated with a step increase in facial muscle activation across the brow and cheek. Group differences were significantly reduced activation in facial muscles during videos considered subjectively negative or rated as high arousal in depressed volunteers compared with controls.

**Conclusion:**

We demonstrate for the first time that it is possible to detect facial expression hypo-reactivity in adults with depression in response to emotional content using glasses-based optomyography sensing. It is hoped these results may encourage the use of optomyography-based sensing to track facial expressions in the real-world, outside of a specialized testing environment.

## Introduction

Depression is a devastating condition characterized by persistent feelings of sadness, hopelessness, and loss of interest. Although appropriate symptom management can help, existing clinical tools for monitoring depression are inadequate. Face-to-face interviews with clinicians are time and resource-intensive and do not permit a fine-grained measurement of day-to-day change in mood symptoms. Self-rated questionnaires such as the Patient Health Questionnaire-9 [PhQ-9; (1)] address some of these issues but still require a high level of engagement by the individual with depression. Because questionnaires capture infrequent snapshots of emotional states, they also fail to track critical details about illness trajectory. Given these limiting factors, there is an explicit need for better ways to monitor depression in mental health care (2).

By measuring the physiological, behavioral and contextual signals relevant to depression, wearables have the potential to address unmet patient needs by providing a symptom management and measurement tool that: (i) does not rely on self-assessment, (ii) can provide immediate feedback about state and trajectory, and (iii) is scalable. The academic community has identified biobehavioural features of depression that could be used for this purpose. Practically, researchers have been limited by the range of sensors available in commercial devices for what can be feasibly measured outside of a specialized environment [for review see: (3)].

Atypical facial expressivity in depression is a good candidate for a measurable biomarker of depressive state (4). However, to date, measurement of facial expressivity has relied on sensors ill-suited to naturalistic monitoring. Facial expressions are traditionally measured using either electromyography (EMG), which measures facial muscle activation directly using electrodes applied to the skin, or using techniques such as Facial Activation Coding (FACs), which measures expression changes (called “action units”) from video footage. In both methods, the zygomaticus muscle in the cheek has been suggested as a reference muscle for positive emotion, and the corrugator supercilii muscle in the brow, a reference muscle for negative emotion (5). It should be noted that the precise correspondence between cheek and brow activation and a specific emotion if measured in real-world conditions outside of a constrained experimental task is both idiosyncratic and highly context-dependent (6).

Laboratory studies measuring facial expressivity in people with depression have typically used mood induction techniques. One method has been to show the volunteer emotionally charged material, either as a video or static image and simply record their reaction. The response of people with depression to viewing positive stimuli is reduced facial muscle activation around the cheek (7, 8). The response to viewing negative stimuli is often [but not always (7)] attenuated facial muscle activation around the brow (9, 10). This is consistent with a context-insensitive hypo-reactivity to emotional stimuli seen in people living with depression (11). In this study we extended these efforts in three novel ways.

First, we measured facial movements during a mood induction task using a new technique called optomyography (12). Optomyography has several advantages over camera-based or EMG-based sensing that make it well-suited to measuring facial expressions in a real-world environment. Unlike camera-based methods, optomyography does not require the face to be staring into a camera or optimal lighting conditions to work. Optomyography also has the advantage of using a fraction of the power of camera-based solutions which makes it a feasible technology for integration into discreet, lightweight eyewear. Like EMG, which is a highly sensitive method to measure valence from facial muscles (5), optomyography is also highly sensitive. Unlike EMG, it does not require electrodes to be attached to the face, conductive gel, or wires. Optomyography measures skin displacement using optical flow algorithms as a proxy for facial muscle activation (13).

Second, we compared sensor outputs to self-reported valence and arousal. Contemporary theories of emotion highlight that momentary feelings of affect are made up of at least two independent dimensions. Valence describes the pleasant–unpleasant dimensions of mood and arousal the relative intensity associated with the affective phenomena (14). Both arousal and valence are widely used in psychological research to describe an individual’s subjective experience and it is the agreed gold standard as a ground truth against which to assess technologies purporting to measure subjective experience (15).

Our hypothesis is that positive valence will be associated with elevated cheek activation and negative valence with elevated brow activation. We also predict that self-reported arousal will be associated with elevated activation from all facial muscles.

Third, we investigated mood induction in adults with depression. Consistent with evidence of attenuated positive and negative emotional reactivity in people living with depression, we hypothesized that relative to the control group, depressed participants would show (i) attenuated cheek activation during videos rated as positive, (ii) attenuated brow activation during videos rated as negative, and (iii) reduced activation across all sensors during videos rated with high levels of arousal.

## Methods

### Participants

Two hundred and five young adults aged between 18 and 40 years were recruited through advertisements placed on social media. Participants were included in the control group if they scored ≤4 on the PhQ9; (1) and ≤4 on the General Anxiety Questionnaire [GAD-7; (16)]. Participants were included in the depressed group if they scored ≥15 on the PhQ9 [moderate-severe depression (1)] and if they met diagnostic criteria for depression using the Mini International Neuropsychiatric Interview V7 [MINI-7; (17)]. Exclusion criteria for all groups were: (a) facial nerve damage which limits the ability to make facial expressions and (b) anatomical constraints that affect glasses fit. Of the 205 recruited, 122 were excluded for GAD-7 or PHQ-9 scores outside of threshold, 6 due to poor glasses fit, 1 due to a technical malfunction during testing and 1 for failing to meet criteria for depression on the MINI. The final sample was 75 participants [38 depressed, 37 healthy controls; mean age (SD) = 25.80 (9.99) years]. In the depressed group, 2 met criteria for major depressive disorder (lifetime), 22 for major depressive disorder (current), 10 for bipolar I disorder (current) and 2 for bipolar disorder I (lifetime) and 2 for bipolar II (lifetime). In the control group, 5 met criteria for major depressive disorder (lifetime) and 1 for bipolar disorder I (lifetime). Participants in the control group did not meet criteria for any other axis 1 mental health disorders assessed using the MINI-7. Volunteers gave informed consent to participate. The NHS research ethics committee reviewed and approved this study (Reference: 22/LO/0415). Clinical Trials identifier: NCT05815459.

### Patient and public involvement

Specific aspects of study design have been directly informed by a lived experience focus group and follow-up surveys. This included the value of the incentives offered and the methodology employed. The study consent and information sheet were reviewed by individuals with lived experience.

### Screening and diagnosis

Screening used the PHQ-9 and GAD-7 and was completed either online or by telephone. The PHQ-9 is a validated screening tool for depression [sensitivity 62%, specificity 96% based on the cut-off score of 15 as used in this study (18)]. A diagnostic assessment was completed using the MINI-7 using DSM-5 criteria. The MINI is a widely used and studied mental health interview tested for clinical and general populations [depression - sensitivity 95%, specificity 84% (19)]. Depression symptoms at the time of testing were measured using a structured version of the Hamilton Depression Rating Scale (20).

### Facial expression detection and mood induction

This study used Emteq’s OCOsense^™^ smart glasses to detect facial muscle activation (13). OCOsense^™^ feature six proprietary OCO^™^ sensors, a 9-axis IMU, altimeter and integrated dual speech detection microphones. The OCOsense^™^ device is highly sensitive and able to extract very precise measurements of skin displacement (21). For our analysis, we focused solely on the data obtained from the OCO^™^ sensors. These sensors use a non-contact optical approach called optomyography (12) to measure skin movement in millimetres in 3 dimensions (X, Y, and Z). The sensors are strategically positioned in the OCOsense^™^ frame to measure displacement resulting from the corrugator muscle (left and right side of the forehead) and zygomaticus major muscle (left and right side of the cheeks). The precise location on the face can be found here (ref). Whilst wearing the glasses, research volunteers were first asked to make 5 repetitions of a smile and frown at their maximal intensity to normalise subsequent outputs. After this, volunteers were asked to passively view 25 short video clips with a 30 s interstimulus interval. The video clips used in this study were sourced from a database of affective videos independently assessed as being either positive, negative or neutral (22). From this database, we selected 9 positive video clips (lasting a combined total of 112.2 s), 10 neutral video clips (lasting a combined total of 250.6 s) and 6 negative video clips (lasting a combined total of 150.6 s). During the interstimulus interval, the research volunteer was asked to report subjective arousal and valence using a 9-point Likert scale. Research volunteers were compensated £50 for their time.

### Pre-processing

The Python programming language was employed for data pre-processing. First linear detrending was performed to remove the influence of long-term sensor drift on measured skin movement. As OCO^™^ sensors measure skin movement in 3 dimensions (X, Y, and Z), the vector magnitude was calculated for each sensor. In this way, we obtained four signals, two representing left and right cheek movement in millimetres, and two representing left and right brow movement in millimetres. To increase the signal-to-noise ratio these signals were smoothed using a rolling median filter with a window size of 15 samples followed by a winsorization transformation using the 99th percentile of the signal to avoid amplitude bursts. After this, person-specific min-max normalization was performed. To do this, the minimum and the maximum values for a smile and frown were extracted from the participant at calibration. Brow and cheek activation whilst watching the videos were normalized to a range between 0 and 1, where 1 is the maximum possible facial expression possible for that participant.

**Figure 1:**
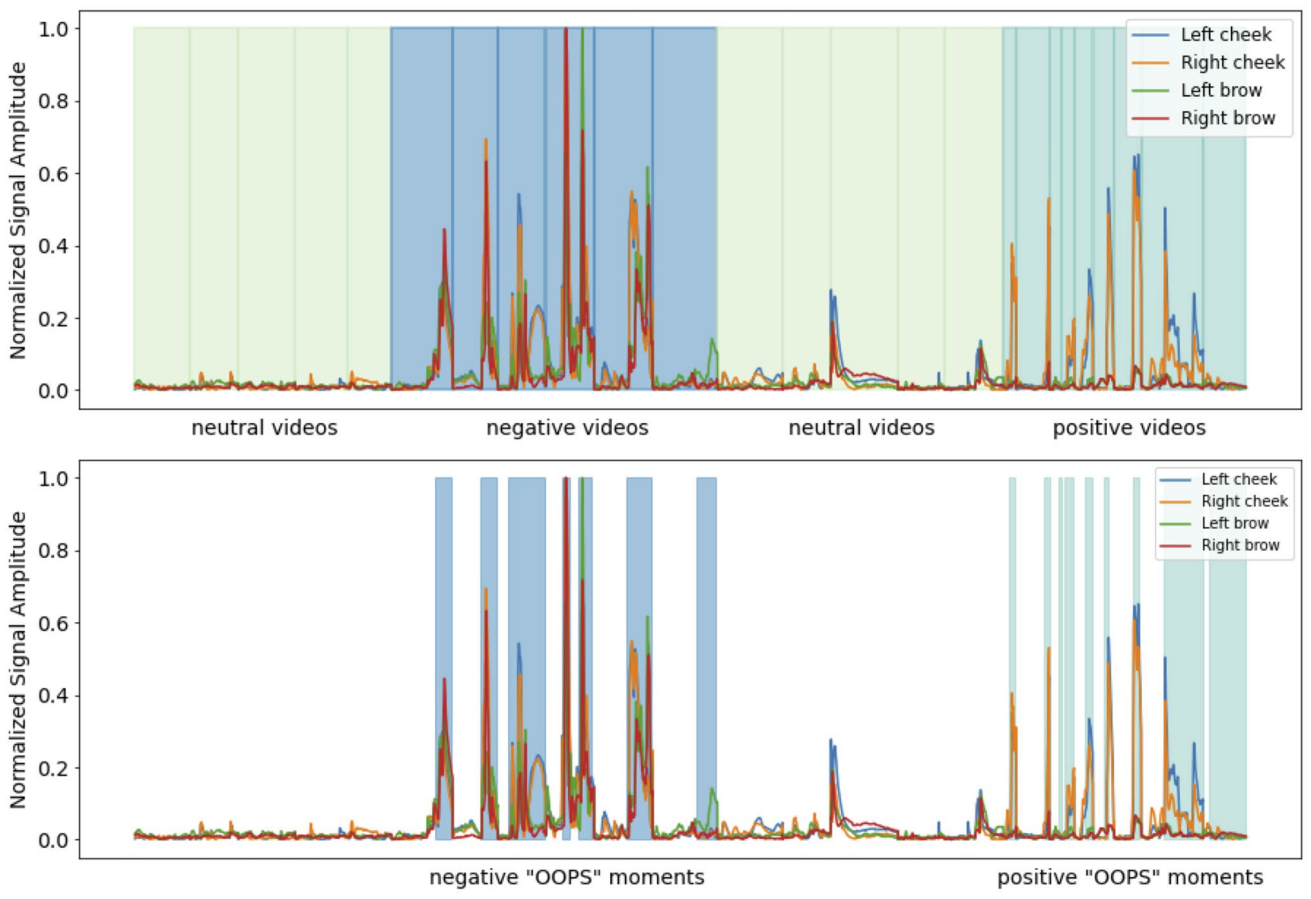
Upper figure. Pre-processed facial muscle activation from a single participant whilst watching affective videos with the interstimulus interval between each video removed. Each video category is colour-coded and with shaded vertical lines indicating the start and end of each clip. Lower figure. The OOPS moments in the negative and positive affective videos.

For each video clip, normalized mean signal amplitude from the left and right brow sensors was extracted at the moment in the video judged to have the highest affective content [the “OOPS” moment (23); Figure 1] as judged by two independent raters. Segmented facial expression data was assigned a label depending on the self-reported level of subjective arousal or valence. Following previous studies (20) if given a subjective arousal or subjective valence rating of between 7 and 9 it was placed into either a high arousal bin or a positive valence bin. If given a subjective rating of between 4 and 6 it was placed into a medium arousal bin or neutral valence bin. If given a subjective rating of between 1 and 3 it was placed into a low arousal bin or a negative valence bin. Five participants were excluded from further tests of valence for not providing any ratings that fell into all three bins (final sample 35HC/34D), 10 participants were excluded from further tests of arousal for not providing any ratings that fell into all three bins (final sample 32HC/32D). Finally, data was square root transformed to meet the normality assumptions of parametric statistics.

### Statistical analysis

SPSS V28 (IMB) was used for statistical analysis. First the normality of residuals assumption was assessed for each through visual inspection of Q–Q plots. Data on facial muscle activation was judged to be normally distributed, self-reported arousal and valence scores were judged to be non-normally distributed. To analyze self-report data, two Friedman tests were performed with either self-reported arousal or self-reported valence as the dependent variable and an identical within-subject variable, video category (three levels: Positive video, Neutral video, and Negative video). Group differences were examined using six Mann–Whitney U tests at *p* < 0.016 Bonferroni correction. To investigate how facial expressions varied by self-reported affect and the presence or absence of depression, we used two 3 × 4 × 2 mixed analyses of variance (ANOVA)s. In the first, the square root of mean signal amplitude from OCOsense glasses sensors was the dependent variable, sensor location on the face (four levels: Left brow, Right brow, Left cheek, Right cheek) and self-rated valence (three levels: Positive, Neutral, Negative) were the within-subject variables and depression status (two levels: Depressed, Healthy controls) the between-subjects variable. In the second, the square root of mean signal amplitude was the dependent variable, sensor location (four levels: Left brow, Right brow, Left cheek, Right cheek) and self-rated arousal (three levels: High, Medium, Low) the within-subject variables and depression status (two levels: Depressed, Healthy controls) the between-group variable. Facial expression data was reported using an uncorrected alpha level of *p* < 0.05.

## Results

### Demographics

There were more female and gender non-binary volunteers in the depressed group (*p* < 0.05). Relative to the control group, the depressed group had elevated depression symptoms (*p* < 0.05). Groups did not differ in age (see Table 1).

**Table 1:** Demographic, clinical, and self-report data.

Clinical assessment/Demographic assessment	Depressed group (*n* = 38) (SD)	Control group (*n* = 37) (SD)	*t*-test/U/*χ*^2^
Age (*y*)	24.52 (4.45)	27.21 (13.71)	1.15
Gender	26F/7M/5NB	25F/12M/0NB	6.32*
Depression symptoms	17.50 (6.31)	1.19 (1.35)	15.39***
Positive videos			
Self-reported Arousal	6.18 (1.39)	6.26 (1.13)	690.5
Self-reported Valence	6.90 (1.20)	6.80 (0.86)	615
Neutral videos			
Self-reported Arousal	6.50 (1.33)	4.05 (0.97)	587.5
Self-reported Valence	4.39 (0.65)	5.16 (0.34)	215.0***
Negative videos			
Self-reported Arousal	6.90 (1.36)	6.93 (1.04)	657.5
Self-reported Valence	2.80 (1.23)	3.02 (0.94)	635

NB, gender non-binary; *p < 0.05 and ***p < 0.001.

### Subjective arousal and valence

For both self-reported arousal [*χ*^2^ (2) = 111.86, *p* < 0.001] and self-reported valence [*χ*^2^ (2) = 132.65, *p* < 0.001] we found a significant main effect of video type. Positive videos were rated with high levels of valence, relative to neutral videos (*T* = 2,841 *p* < 0.001, *r* = 0.83), and neutral videos rated more positively than negative videos (*T* = 55, *p* < 0.001, *r* = 0.82). Negative videos were rated as more arousing than positive videos (*T* = 2,238, *p* < 0.001, *r* = 0.83), and positive videos were more arousing than neutral videos (*T* = 47.50, *p* < 0.001, *r* = 0.53). Volunteers in the depressed group provided lower valence scores for neutral videos than the control group (Table 1).

### Self-rated valence and facial muscle changes

**Figure 2:**
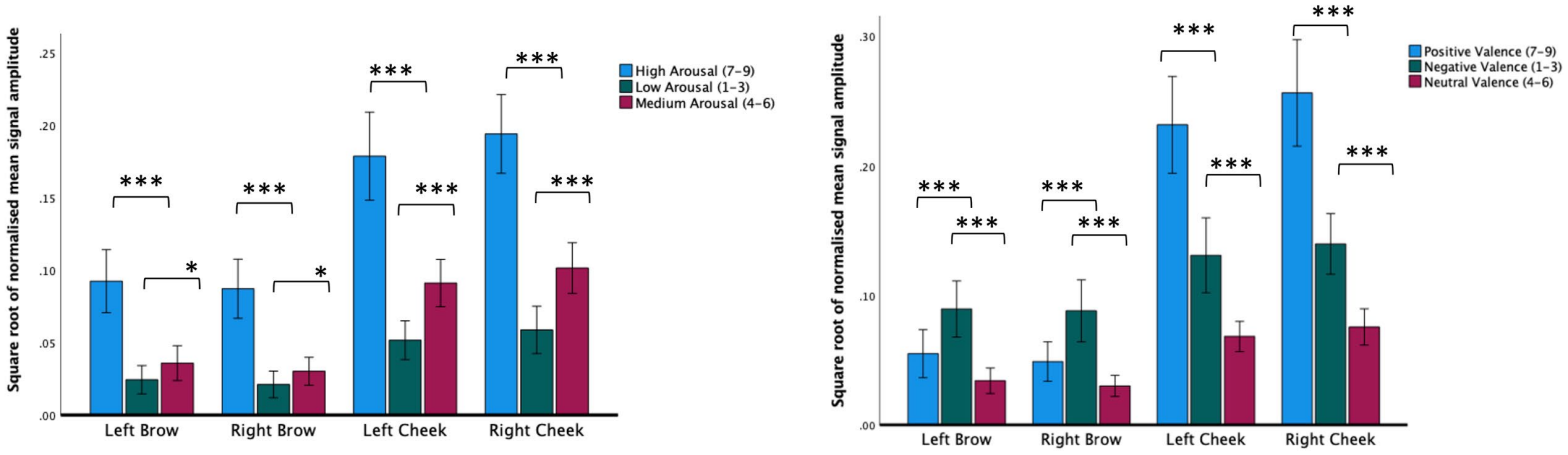
Normalized mean signal amplitude for self-report ratings of arousal (left figure) or arousal (right figure). Within group contrasts report paired samples *t*-tests. Error bars report CI. *p < 0.05, ***p < 0.001.

Investigating sensor outputs as the dependent variable, we found a significant main effect of sensor location on the face (*F*_(1.74,67)_ = 102.57, *p* < 0.001, *r* = 0.78), a significant main effect of the self-rated valence of the video being watched (*F*_(1.15,67)_ = 43.48, *p* < 0.001, *r* = 0.38) and a significant valence × location interaction (*F*_(2.37,67)_ = 79.71, *p* < 0.001, *r* = 0.50). Subjective ratings of between 7 and 9 (positive) were associated with increased left and right cheek activation compared to videos given subjective ratings of between 4 and 6 (neutral) or subjective ratings of between 1 and 3 (negative). Negative video clips were associated with increased left and right brow activation compared with clips rated as either positive or neutral (see Figure 2; Table 2).

**Table 2:** Within-group contrasts for video-based mood induction based on self-reported valence and arousal.

Muscle	Mood contrast	*t*-test	Effect size (*r*)
Valence			
Left brow	Positive – Neutral	2.25*	0.07
	Positive – Negative	3.94***	0.19
	Negative – Neutral	6.71***	0.39
Right brow	Positive – Neutral	2.33*	0.07
	Positive – Negative	4.08***	0.20
	Negative – Neutral	6.59***	0.38
Left cheek	Positive – Neutral	12.75***	0.69
	Positive – Negative	6.63***	0.39
	Negative – Neutral	5.60***	0.30
Right cheek	Positive – Neutral	13.41***	0.71
	Positive – Negative	6.21***	0.36
	Negative – Neutral	6.13***	0.35
Arousal			
Left brow	High – Medium	7.20***	0.42
	High – Low	9.81***	0.59
	Low – Medium	4.96***	0.26
Right brow	High – Medium	7.82***	0.46
	High – Low	10.32***	0.61
	Low – Medium	4.67***	0.23
Left cheek	High – Medium	8.65***	0.51
	High – Low	9.93***	0.59
	Low – Medium	5.79***	0.32
Right cheek	High – Medium	10.40***	0.60
	High – Low	11.38***	0.66
	Low – Medium	6.11***	0.35

*p < 0.05 and ***p < 0.001.

**Figure 3:**
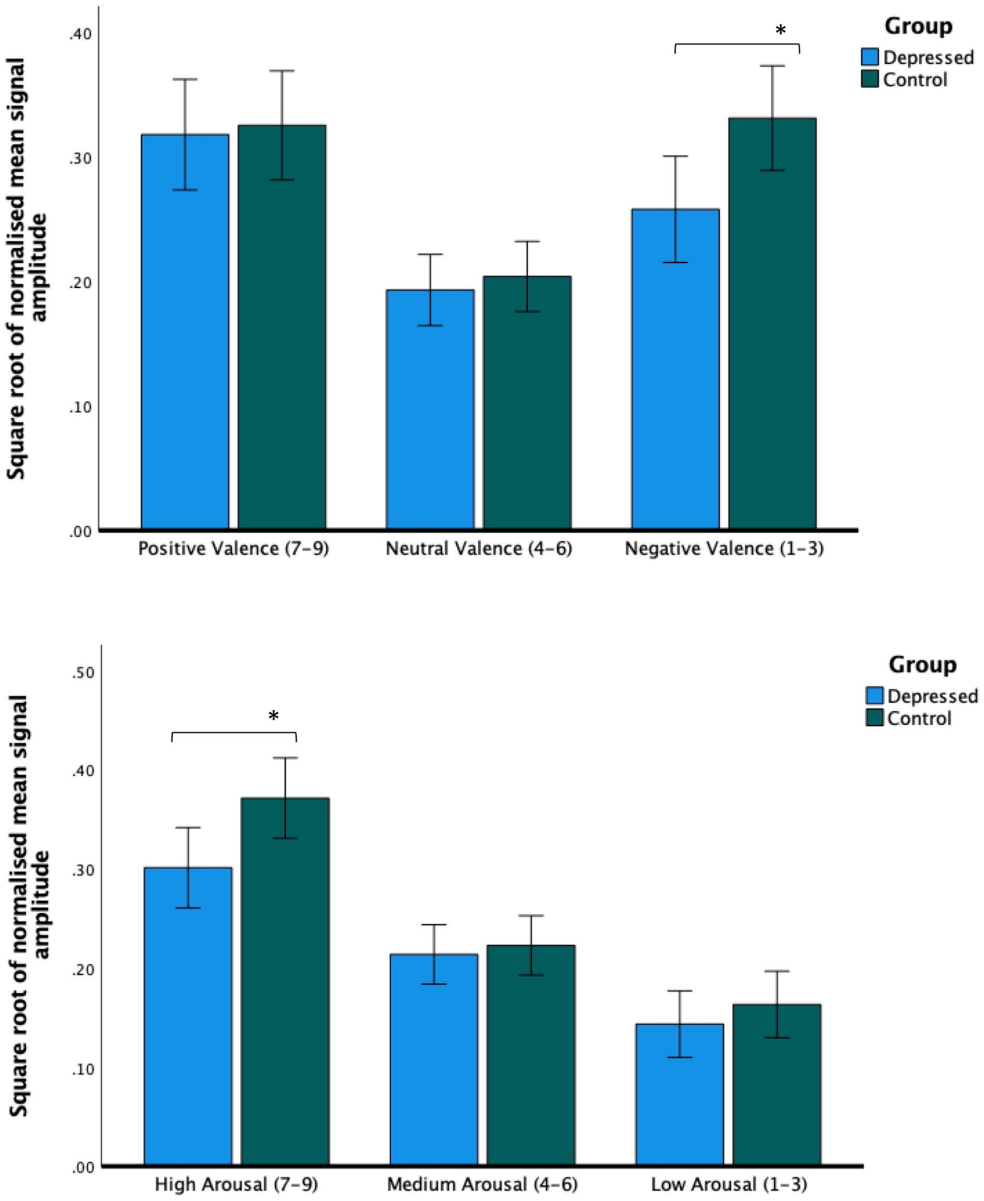
Facial expression intensity from averaged bilateral cheek and brow sensors. Across all sensors, people with depression made less intense facial expressions during videos rated as being negatively valenced (upper figure) and videos rated as highly arousing (lower figure). *Post hoc* tests report paired independent samples *t*-tests. Error bars report CI. *p < 0.05.

There was no main effect of depression status, (*F*_(1,67)_ = 1.8, *p* = 0.18, *r* = 0.04), but there was a significant valence × group interaction (*F*_(1.82,67)_ = 3.58, *p* = 0.035, *r* = 0.10). Relative to the control group, the depressed group made less intense facial expression responses when presented with stimuli self-reported as negative (*t*_(67)_ = 2.58, *p* = 0.012, *r* = 0.30; see Figure 3).

### Self-rated arousal and facial muscle changes

Using sensor data as the dependent variable, we found a significant main effect of self-reported arousal (*F*_(1.69,62)_ = 110.32, *p* < 0.001, *r* = 0.57), a significant main effect of the location of the sensor on the face (*F*_(1.46,62)_ = 81.93, *p* < 0.001, *r* = 0.77) and a significant location × arousal interaction (*F*_(2.10,62)_ = 4.78, *p* = 0.006, *r* = 0.10). Across all facial muscles, there was a step increase in facial muscle activation depending on subjective ratings. Subjective arousal ratings of between 7 and 9 (high) were associated with elevated brow and cheek activation relative to videos given subjective ratings between 4 and 6 (medium) or 1 and 3 (low). Likewise, videos given subjective arousal scores of between 4 and 6 were associated with increased brow and cheek facial muscle activation relative to those rated as low arousal (see Figure 2; Table 2).

There was no significant main effect of depression (*F*_(1,62)_ = 2.70, *p* = 0.12, *r* = 0.08). There was a significant arousal × group interaction (*F*_(1.63,62)_ = 3.41, *p* = 0.04, *r* = 0.08). Relative to the control group, the depressed group made less intense facial muscle responses when viewing videos rated as being highly arousing (*t*_(62)_ = 2.4, *p* = 0.019, *r* = 0.29; see Figure 3).

### Sensitivity analyses

To explore possible effects of comorbid generalized anxiety disorder and bipolar disorder diagnoses, between groups contrasts were repeated excluding volunteers who met criteria for either condition. Excluding these volunteers did not affect the direction or significance of any results (all *p* values *p* < 0.05). To explore possible confounding effects of gender, across all populations we ran separate between groups *t*-tests with gender as the between subjects variable (male, female, Non-binary) and mean signal amplitude in the left cheek, right cheek, right brow, left brow and average movement across all facial muscles the between subjects variable. Males showed context-unspecific activation in the left and right cheek only (left cheek; *t* = 3.26, *p* = 0.006 and right cheek; *t* = 2.79, *p* = 0.03). There were no other significant effects.

## Discussion

Clinicians have long observed the reduction in facial movements in depression [for citations see: (24)]. These clinical observations were subsequently verified by objective means using facial EMG and more recently computer vision. These techniques are not suitable for continuous, home-based monitoring. Glasses are a common wearable technology that will soon follow the pathway of the watch and become sensor-enabled for health-based well-being monitoring.

In this study we sought to investigate whether optomyography-based sensing in glasses can be used to measure self-reported changes in emotional state after volunteers watch video clips judged to be positive, neutral or negative. We also sought to investigate how state changes may be moderated by the presence or absence of depression. There are several findings of note.

First, as predicted, we found that mean signal amplitude varied significantly depending on the affective content of the video being watched. Videos perceived as positive were associated with increased left/right cheek activation compared with videos perceived as subjectively neutral or subjectively negative. Videos perceived as subjectively negative were associated with increased brow activation compared with videos perceived as subjectively positive or subjectively neutral. We also found activation of both brow and cheek muscles elevated during videos perceived as highly arousing. The association between a smile and positive valence and a frown and negative valence is by now very well established (5). Likewise, a “V-shaped” relation between arousal and valence, where highly negative and highly positive material are both associated with increased facial muscle activation is similarly well documented (25). These findings confirm that optomyography can detect facial expression-derived affective responses to positive, negative and neutral video footage.

Second, as predicted, we found that people with depression made less intense facial muscle activations during material judged to be subjectively negative. Interestingly, a similar effect was not observed when volunteers viewed positive stimuli. Furthermore, and against expectations, we observed hypoactivation from all sensors across the brow and cheek. Although a reduction in overall facial expressivity to negative stimuli is consistent with previous studies (10, 26), the trend in the literature appears to be that people with depression smile less when viewing positive stimuli [for discussion see: (27)]. One explanation for this discrepancy is that it is not the total intensity of expressions that is important *per se*, but the properties of and interactions between different expressions. For example, Reed, Sayette and Cohen (28) reported that depressed volunteers smiled just as frequently as non-depressed controls when viewing comedy clips, but were more likely to blend expressions, interpreted to signify sadness. Following a review of the literature, Scherer et al. (27) suggests recording expression intensity or frequency may obfuscate variation in expression duration and acceleration, which may be more predictive of depression [see also (4)]. Although developing techniques to measure expression dynamics using optomyography goes beyond the scope of this paper, it represents an important avenue of future research.

Third, we found that mean signal amplitude across facial muscle sensors were reduced in people with depression relative to controls during high-arousal videos. This is the first time a V-shaped relationship between facial expressions and subjective arousal and valence was investigated in depressed individuals. However, our results are supported by wider evidence of blunted physiological reactivity to positive and negative stimuli as assessed using alternative sensors known to measure arousal, including heart rate, blood pressure, startle magnitude and skin conductance (11). Arousal has been hypothesized to moderate how the affective quality of an experience is perceived, thus regulating valence changes diachronically. As suggested by Petrolini and Viola (25), low arousal in depression may underlie a greater difficulty in moving along the valence spectrum. This has been proposed to present as emotional rigidity. They predicted that people with depression would show a reduced response to the affective qualities of a stimulus, consistent with what we observed.

This study has several strengths. This includes a moderate sample size, robust diagnostic procedures and a validated methodology. However, there are limitations that need to be considered. First, depression was not subtyped in this study. The impact of an anxious depressed subtype or a melancholic subtype on emotional and physiological responses is unclear and requires evaluation. Second, consistent with previous studies, we measured facial reactions over a relatively short duration under relatively contrived conditions. For facial expressions to be ultimately used for health-based monitoring, future studies will need to explore the generalizability of these findings, this may include a test–retest procedure and an exploration into how tolerated use of the glasses are over extended periods of wear. Third, we found that normalized facial muscle activation was elevated in males. This result is not consistent with the wider literature and may be due to the small sample size employed (29).

In summary, we demonstrate that optomyography-based sensing could have utility for naturalistic real-world recording of facial expressivity, without the need to apply electrodes or use a face-facing camera and good lighting conditions to do so. Furthermore, we show for the first time using sensor-enabled glasses that people with depression show reduced emotional reactivity to evocative stimuli. This is a partial replication of previous studies and heralds the potential of moving mental health assessment from being episodic and subjective to being continuous and objective. We ultimately envisage two potential applications for the device. The first involves its on-demand use, specifically for conducting targeted tests. The second application entails utilizing the device in everyday life, enabling clinicians and patients to access real-life data.

## Data availability statement

The raw data supporting the conclusions of this article will be made available by the authors, without undue reservation.

## Ethics statement

The NHS research ethics committee reviewed and approved this study (Reference: 22/LO/0415). The studies were conducted in accordance with the local legislation and institutional requirements. The participants provided their written informed consent to participate in this study.

## Author contributions

HG, CN, SC, IM, IK, LS, and MG contributed to the first draft which was written by MB. Data collection and quality control was completed by IK, IM, LS, SC, and MB. The study was designed by MB and SS. MG designed the analysis plan and IK and MB analyzed the data. CN is the guarantor. All authors contributed to the article and approved the submitted version.

## Conflict of interest

CN, HG, IM, MB, SS, IK, LS, DB, and SC worked for Emteq Ltd. who manufactured the OCOsense glasses used in this study. JS has been principal investigator or sub-investigator on studies sponsored by Takeda, Janssen and Lundbeck Plc.

The remaining author declares that the research was conducted in the absence of any commercial or financial relationships that could be construed as a potential conflict of interest.The authors declare that this study received funding from Emteq Labs. The funder was involved in the study design, collection, analysis, interpretation of data, the writing of this article, and the decision to submit it for publication. The funding was awarded to Emteq Ltd. by Innovate UK as part of an innovation in precision medicine funding award (ref: 105207).

## Publisher’s note

All claims expressed in this article are solely those of the authors and do not necessarily represent those of their affiliated organizations, or those of the publisher, the editors and the reviewers. Any product that may be evaluated in this article, or claim that may be made by its manufacturer, is not guaranteed or endorsed by the publisher.
